# Assessing diagnostic tests for shrimp allergy in children: A multicenter trial

**DOI:** 10.1016/j.jacig.2025.100471

**Published:** 2025-04-10

**Authors:** Yuri Takaoka, Yuki Tsurinaga, Yukiko Hiraguchi, Masaaki Hamada, Atsuko Nakano, Tomoko Kawakami, Ikuo Okafuji, Nayu Iwakoshi, Masaaki Doi, Keita Otsuka, Yukiko Sugimoto, Norihito Iba, Junko Kumon, Rumi Ueno, Tamana Nakano, Tomohiro Yamaguchi, Yohei Fuksawa, Amane Shigekawa, Yukinori Yoshida, Makoto Kameda

**Affiliations:** a Department of Pediatrics, Osaka Prefectural Hospital Organization Osaka Habikino Medical Center, Habikino, Osaka, Japan; b Department of Pediatrics, Osaka Saiseikai Nakatsu Hospital, Shibata, Kita, Osaka, Japan; c Department of Pediatrics, Yao Municipal Hospital, Ryuge, Yao, Osaka, Japan; d Department of Pediatrics, Kokuho Chuo Hospital, Daimonjimiyako, Tawaramoto, Shiki, Nara, Japan; e Department of Pediatrics, Sumitomo Hospital, Nakanoshima, Kita, Osaka, Japan; f Department of Pediatrics, Kobe City Hospital Organization, Kobe City Medical Center General Hospital, Minatoshima, Chuo, Kobe, Hyogo, Japan; g Department of Pediatrics, Higashiosaka City Medical Center, Nishiiwata, Higashiosaka, Osaka, Japan; h Department of Pediatrics, Nara City Hospital, Higashikidera, Nara, Japan; i Department of Pediatrics, Hoshigaoka Medical Center, Hoshigaoka, Hirakata, Osaka, Japan; j Minnanokodomo Allergy Clinic, Tomoda, Wakayama, Japan

**Keywords:** Shrimp hypersensitivity, IgE, skin prick test (SPT), ROC-curve analysis, tropomyosin, allergic reaction symptoms

## Abstract

**Background:**

Clinical research on pediatric shrimp allergy is limited.

**Objective:**

We sought to evaluate the diagnostic accuracy and safety of testing methods for shrimp allergy.

**Methods:**

An oral food challenge (OFC) for shrimp was conducted on Japanese children with suspected shrimp allergy. Before the OFC, shrimp-, tropomyosin-, house dust mite–, and cockroach-specific IgE levels were measured, along with skin prick tests (SPTs). OFC results using epinephrine as a safety indicator determined persistent, mild, or tolerant shrimp allergy.

**Results:**

Sixty-six children (median age, 6 years) underwent the OFC. All patients demonstrated house dust mite–specific IgE level exceeding 0.35 IUA/mL. Sixteen were diagnosed with persistent shrimp allergy, defined by Anaphylaxis Scoring Aichi scores greater than or equal to 10 or scores of 5 with urticaria. A 15-year-old required epinephrine for anaphylaxis. Eight children with negative results (scores ≤ 9) reported mild symptoms after repeated home ingestion following the OFC. Median SPT wheal diameters in persistent, mild allergic, and tolerant groups were similarly elevated (8.5 vs 9.5 vs 8.0 mm; *P* = .99). Patients with persistent shrimp allergy had higher median shrimp- and tropomyosin-specific IgE level than those classified as mild or tolerant (shrimp: 73.5 vs 30.0 vs 9.4 IUA/mL; *P* = .01; tropomyosin: 68.0 vs 41.9 vs 11.5 IUA/mL; *P* = .16). Receiver-operating characteristic analysis determined optimal IgE cutoff values as 58.2 IUA/mL for shrimp-specific IgE and 33.5 IUA/mL for tropomyosin-specific IgE.

**Conclusions:**

SPT showed limited symptom correlation, whereas shrimp-specific IgE demonstrated greater diagnostic value than tropomyosin-specific IgE. No IgE cutoff accurately predicts a successfully passed OFC.

Food allergies are a significant public health concern in pediatric populations in Japan, with shrimp allergy ranking as the seventh most common allergy among Japanese children.^1^ Although research indicates that shrimp-induced anaphylaxis occurs less frequently in children than in adults,^2^ limited clinical studies have specifically addressed the pediatric presentation of shrimp allergy. In a cohort study of 99 Japanese individuals with shrimp allergy (mean age, 19.5 years; children aged 1-6 years: n = 29), anaphylaxis was reported in 61.6% of cases; however, pediatric-specific rates were not provided.^3^

Tropomyosin is recognized as the primary sensitizing allergen in shrimp,^4^ and the World Health Organization and the International Union of Immunological Societies have acknowledged 9 shrimp allergens.^5^ A study by Pascal et al^6^ demonstrated the roles of tropomyosin and sarcoplasmic calcium-binding proteins in mediating clinical reactivity to shrimp.

In addition, shrimp allergy exhibits cross-reactivity with dust mite allergy, known as “mite-shrimp allergy syndrome,” in which affected individuals experience oral symptoms without systemic involvement.^7^

However, limited data exist regarding the association between clinical manifestations following shrimp ingestion and shrimp-specific IgE levels or skin prick test (SPT) reactivity in pediatric patients.

This study aimed to investigate the clinical features of shrimp allergy through oral food challenges (OFCs) in Japanese children with suspected shrimp hypersensitivity. In addition, we evaluated correlations between clinical symptoms, IgE titers specific to shrimp and tropomyosin, and SPT outcomes to shrimp.

## Methods

### Diagnosis procedure

We conducted an OFC for Japanese children with suspected shrimp allergy, adhering to a standardized multicenter protocol at Kinki Food Challenge Network facilities. Children aged 0 to 15 years, whose parents or legal guardians provided informed consent, were eligible if they demonstrated elevated shrimp-specific IgE antibody levels or had a history of allergic symptoms following shrimp ingestion between 2019 and 2022. Exclusion criteria encompassed poorly controlled allergic comorbidities, such as asthma and atopic dermatitis.

Before the OFC, each participant underwent blood testing and SPT. During the OFC, children were administered boiled black tiger shrimp (*Penaeus mondon*) or white-leg shrimp (*Litopenaeus vannamei*) in portions of 2, 5, or 10 g every 30 minutes, totaling 17 g (about 2.5-3.0 medium shrimps) within 1 hour. On the basis of the Standard Tables of Food Composition in Japan 2020 (eighth revised edition), 17 g of cooked white-leg shrimp (tempura) contains 3400 mg of protein, which was approximated as the protein content in 17 g of boiled shrimp used in the OFC.^8^ Patients were then observed for a minimum of 2 hours postingestion to assess for symptoms.

Symptom severity was scored using the Anaphylaxis Scoring Aichi (ASCA) system,^9^ as detailed in Table E1 and Fig E1 (in the Online Repository available at www.jaci-global.org). The ASCA system evaluates allergic responses across 5 organ systems (dermatologic, respiratory, gastrointestinal, cardiovascular, and neurologic), with each system receiving a score from 0 to 60 on the basis of symptom severity. The total ASCA score (maximum of 240 points) represents the sum of individual organ scores.

A positive OFC result, indicating persistent shrimp allergy, was defined by a total ASCA score of 10 or more points or by the presence of objective dermatologic symptoms (eg, urticaria scored at ≥5 points). Patients with positive OFC results were categorized as having persistent shrimp allergy. Abdominal pain was assessed using a face pain scale (FS) from 0 to 4. Positive abdominal pain was defined by observable symptoms, such as vomiting and diarrhea, or an FS score of 2 or higher; an FS score of 1 alone was not considered positive.

An OFC was deemed negative if the ASCA total score was less than 9 points, excluding local urticaria scored at 5 points in the dermatologic category. Cases with only oral irritation were assigned an ASCA score of 1 point and classified as negative. Patients with negative OFC results were advised to continue shrimp ingestion at home without restrictions on physical activity, and their shrimp allergy status was reassessed in an outpatient setting within 3 months post-OFC.

Symptoms observed during continued home ingestion were classified as allergic reactions, whereas the absence of symptoms was classified as tolerance.

### Skin prick test

An SPT was performed before the OFC using a bifurcated needle (Tokyo M. I. Co, Tokyo, Japan) with shrimp extract (Allergen Scratch Extract “Torii Shrimp”, Torii Pharmaceutical Co, Ltd, Tokyo, Japan). The mean wheal diameter was measured after 15 minutes and compared with negative and positive controls (10 mg/mL histamine dihydrochloride). A wheel diameter of 3 mm or higher was considered a positive response.

### Blood testing

Shrimp-specific, tropomyosin-specific, house dust mite (*Dermatophagoides pteronyssinus* and *Dermatophagoides farinae*)–, and cockroach-specific IgE antibody levels were measured using the Ala STAT 3g system (Siemens Healthcare, Erlangen, Germany) before the OFC. Values of 0.35 IUA/mL or higher were considered positive.

### Primary end point

The primary end point was the identification of persistent shrimp allergy through the OFC.

### Statistical analysis

All analyses were conducted using IBM SPSS Statistics version 29 (IBM, Armonk, NY). The chi-square test was used to compare categorical variables, such as sex, medical history, and other allergic conditions, across diagnostic categories (confirmed persistent shrimp allergy, mild shrimp allergy, and tolerance). The Kruskal-Wallis test was used to evaluate differences in age, IgE levels, and SPT wheal diameters. Statistical significance was set at a 2-tailed *P* value less than .05. Receiver-operating characteristic (ROC) curve analysis was used to identify optimal cutoff values for shrimp- and tropomyosin-specific IgE levels predictive of positive OFC outcomes. The area under the curve (AUC) was calculated for each allergen to assess diagnostic performance, with higher AUC values indicating improved diagnostic accuracy. Sensitivity and specificity were calculated at the cutoff values to evaluate test effectiveness in differentiating allergic from nonallergic individuals.

### Ethical considerations

This study was approved by the Research Ethics Committee of Osaka Habikino Medical Center (approval no. 932-5). Written informed consent was obtained from the parents or legal guardians of all participating children.

## Results

### Patient characteristics

A total of 67 patients were enrolled, of whom 66 (42 male) completed the OFC. Demographic data are provided in Table I. The median allergen-specific IgE levels were as follows: shrimp, 16.5 IUA/mL; tropomyosin, 16.4 IUA/mL; *D pteronyssinus*, 44.5 IUA/mL; *D farinae*, 80.6 IUA/mL; and cockroach, 2.7 IUA/mL. All patients exhibited house dust mite–specific (either *D pteronyssinus* or *D farinae*) IgE levels greater than 0.35 IUA/mL. Median SPT wheal diameter was 8.5 mm. Forty-three patients (65%) had a symptomatic history of shrimp allergy, whereas 23 patients (35%) showed elevated shrimp-specific IgE levels without any symptomatic history.

**Table I:** Baseline characteristics of patients who underwent the OFC test

Variable	Patients with suspected shrimp allergy (n = 66)
Age (y), median (range)	6 (1-15)
Sex: male, n (%)	42 (63.6)
Past history of symptoms by shrimp, n (%)	42 (63.6)
Shellfish allergy except shrimp,∗ n (%)	12 (18.2)
Bronchial asthma, n (%)	17 (25.8)
Atopic dermatitis, n (%)	43 (65.1)
Allergic rhinitis, n (%)	33 (50.0)
Allergen-specific IgE (Ala STAT) (IUA/mL), median (range)	
Shrimp	16.5 (0.1-265)
Tropomyosin (Pen m 1)	16.4 (0.1-336)
*Dpteronyssinus*	44.5 (0.2-497)
*Dfarinae*	80.6 (0.4-500)
Cockroach	2.7 (0.1-72.0)
Wheal diameter of shrimp SPT (mm), median (range)	8.5 (0-32.5)

∗ Patients who had symptoms by shellfish other than shrimp.

### Diagnosis of shrimp allergy via OFC

Following the OFC, 16 patients were diagnosed with persistent shrimp allergy, whereas 50 had negative OFC outcomes. These OFC-negative patients were instructed to continue shrimp ingestion at home. Of these, 40 were confirmed as tolerant, exhibiting no symptoms upon repeated shrimp ingestion, whereas 8 experienced mild symptoms, leading to a diagnosis of mild shrimp allergy. Among these 8, 1 reported dermatologic symptoms, 1 abdominal pain, and 6 oral symptoms. Two patients declined further home ingestion, preventing a definitive diagnosis. Characteristics of each diagnostic category are provided in Table II. Among the 43 patients (65%) with symptomatic histories of shrimp allergy, 13 were diagnosed with persistent allergy, 6 with mild allergy, and 24 as tolerant.

**Table II:** Comparison of characteristics among patients with persistent, mild allergic, and tolerant shrimp allergy

Variable	Oral shrimp challenge test result			*P* value
	Positive	Negative		
	Persistent (n = 16)	Mild allergic (n = 8)	Tolerant (n = 40)	
Age (y), median (range)	7 (3-15)	7 (3-11)	6 (1-14)	.49
Sex: male, n (%)	10 (62.5)	5 (62.5)	21 (52.5)	.74
Past history of symptoms by shrimp, n (%)	12 (75.0)	7 (87.5)	24 (60.0)	.24
Shellfish allergy except shrimp, n (%)	3 (18.8)	1 (12.5)	8 (20.0)	.88
Bronchial asthma, n (%)	6 (37.5)	1 (12.5)	9 (22.5)	.34
Atopic dermatitis, n (%)	13 (81.3)	3 (37.5)	23 (57.5)	.09
Allergic rhinitis, n (%)	11 (68.8)	4 (50.0)	18 (45.0)	.27
Allergen-specific IgE (Ala STAT) (IUA/mL), median (range)				
Shrimp	73.7 (0.3-265.0)	30.0 (4.2-63.9)	9.4 (0.1-213.0)	.01
Tropomyosin (Pen m 1)	68.0 (0.2-336.0)	41.9 (2.5-84.6)	11.5 (0.1-288.0)	.16
*Dpteronyssinus*	53.0 (0.2-497.0)	23.1 (1.2-141.0)	37.1 (0.5-253.0)	.46
*Dfarinae*	118.5 (3.4-500.0)	70.2 (1.1-206.0)	64.4 (0.4-378.0)	.44
Cockroach	4.4 (0.1-72.0)	4.5 (0.3-13.6)	1.8 (0.1-36.2)	.38
Wheal diameter of shrimp SPT (mm), median (range)	8.5 (4.0-23.5)	9.5 (4.5-32.5)	8.0 (0-22.5)	.99
Total dose of oral shrimp challenge test (g),∗ median (range)	12 (2-17)	—	—	—

This table provides comparison of the details of shrimp persistent, mild allergic, and tolerant patients. *Persistent* refers to patients with a positive shrimp OFC. *Mild allergic* denotes patients with negative shrimp OFC results and mild symptoms following shrimp ingestion at home after OFC. *Tolerant* describes patients with negative shrimp OFC results and no subsequent symptoms after shrimp ingestion at home after OFC.

∗ Total dose of mild allergic and tolerant groups was 17 g of shrimp. Categorical data, including sex, medical history, and other allergic diseases, were compared across 3 diagnostic categories using the χ^2^ test. The Kruskal-Wallis test was performed to compare age, specific IgE levels, and SPT wheal diameters.

### OFC-positive cases

Among the 16 OFC-positive patients (Table Ⅲ), the median ASCA score was 13 points, indicating mild reactions in most cases; 4 patients presented with severe reactions (scores of 30 and 70 points). A 15-year-old patient developed anaphylaxis after consuming 2 g of shrimp, involving cardiovascular and gastrointestinal symptoms, necessitating intramuscular epinephrine administration.

**Table III:** Characteristics of persistent shrimp allergy cases

No.	Age (y)	Oral shrimp challenge test				SPT∗ (mm)	Allergen-specific IgE (Ala STAT) (IUA/mL)				
		Total dose (g)	Total score of ASCA	Symptoms†	Time to symptoms appearance,‡ (min)	Shrimp	Shrimp	Tropomyosin (Pen m 1)	*D pteronyssinus*	*D farinae*	Cockroach
1	3	2	15	S(5),R(10)	6	18	0.3	0.2	0.2	3.4	0.1
2	5	7	10	G(10),O(1)	10	5	26.9	14.9	112	186	2.9
3	5	7	5	S(5)	98	12	86.9	133	118	252	20.5
4	5	17	11	G(11),O(1)	0	7	4.4	0.1	17.2	3.9	0.3
5	6	7	10	R(10)	7	10	59.2	52.7	27.2	133	1.7
6	6	2	20	S(10),R(5),N(5)	10	9	265	336	497	500	72
7	6	17	10	G(5),S(5)	10	6	10.1	0.1	38.7	104	0.1
8	7	7	6	O(1),S(5)	2	7.5	77	39.3	8.5	13.5	5.7
9	7	17	10	O(1),R(10)	1	9	13.9	4.7	0.4	0.4	0.3
10	7	17	30	G(20),O(1),N(10)	0	8.5	80.4	106	67.3	64.6	15.6
11	8	17	25	G(10),O(1),R(10), S(5)	10	5	144	170	95.7	367	27
12	9	17	35	G(20),O(1),S(5),N(10)	10	23.5	3.3	0.4	188	416	0.7
13	9	17	30	G(20),O(1),N(10)	0	8.5	111	192	158	185	22.5
14	11	17	15	G(5),O(1),S(5),N(5)	10	5.5	132	133	208	213	16.1
15	13	2	11	O(1),R(10)	0	4	125	121	15.5	18.6	3.1
16	15	2	70	G(10),O(1),R(20),C(40)	2	11.5	70.3	83.3	24.7	41.4	9.2

This table provides details of the patients who tested positive in the oral shrimp challenge test.

*C*, Cardiovascular symptoms; *G*, gastrointestinal symptoms; *N*, neural symptoms; *O*, oral symptoms; *R*, respiratory symptoms; *S*, skin symptoms.

∗ Mean wheal diameter.

† The score for each organ is indicated in parentheses. The score for oral symptoms is included under gastrointestinal symptoms in the ASCA system (see Table E1). The total score is the sum of each organ’s score.

‡ The time of 0 min indicates that symptoms occurred during ingestion.

Gastrointestinal symptoms were the most common, reported by 9 patients (56%), followed by skin (50%), respiratory (44%), neurologic (31%), and cardiovascular (6%) symptoms. In addition, 11 patients (69%) experienced oral symptoms. The median cumulative dose of shrimp consumed during the OFC was 12 g. The median time to symptoms appearance after ingestion was 6.5 minutes. All but 1 patient experienced symptoms within 10 minutes, and 4 patients reported symptoms during ingestion.

### Shrimp- and tropomyosin-specific IgE levels

Of the 66 patients, 16 were diagnosed with persistent shrimp allergy (OFC-positive), 8 had mild allergies (on the basis of home ingestion symptoms), and 40 were classified as tolerant. Median shrimp-specific IgE levels in these groups were 73.7, 30.0, and 9.4 IUA/mL, respectively, with a statistically significant difference across groups (*P* = .013; Table Ⅱ). Tropomyosin-specific IgE levels showed median values of 68.0, 41.9, and 11.5 IUA/mL for the persistent, mild, and tolerant groups, respectively, although no significant intergroup difference was observed (*P* = .160).

**Fig 1:**
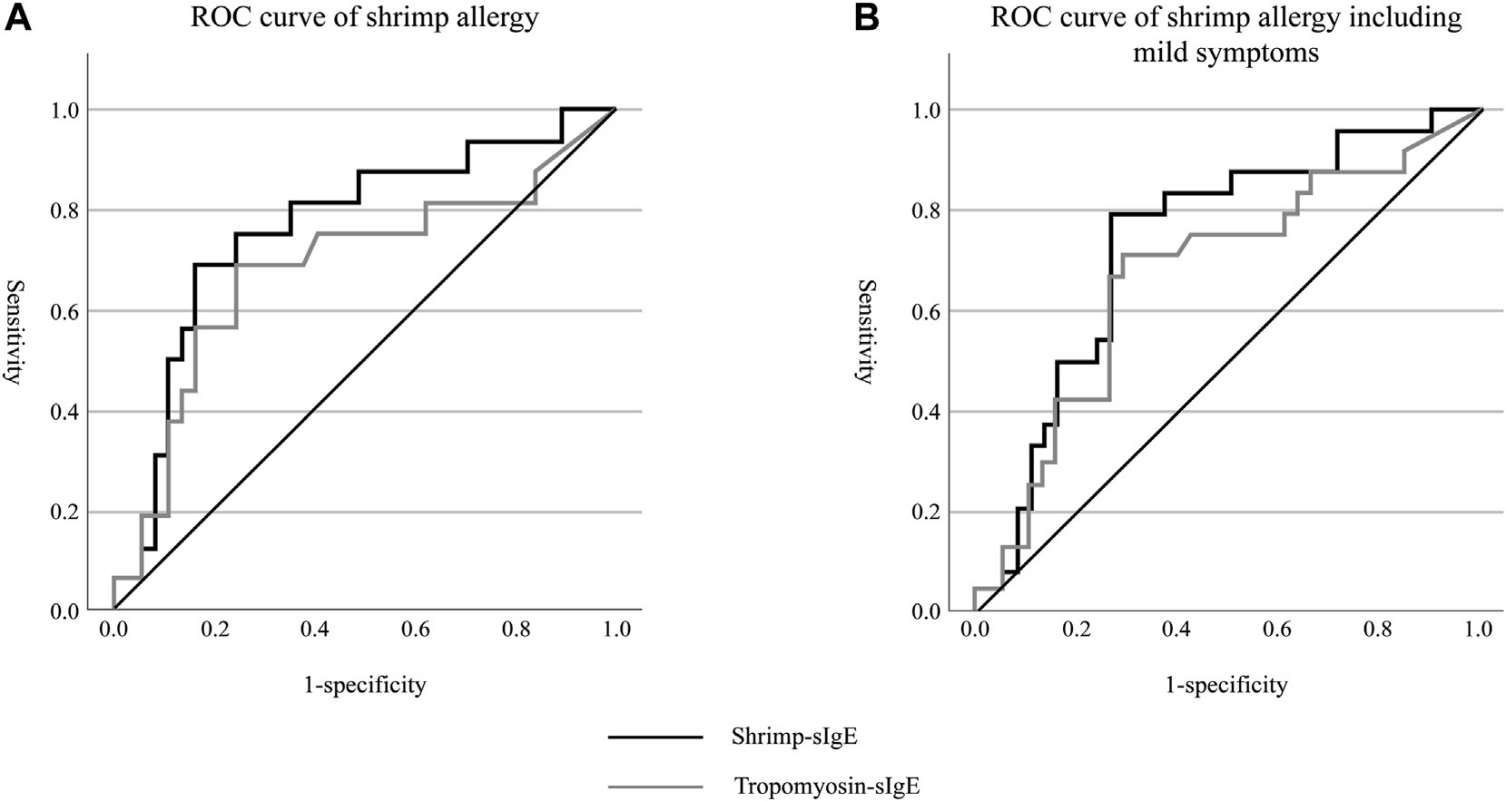
ROC curves of shrimp- and tropomyosin-specific IgE. The *black line* represents shrimp-specific IgE, and the *gray line* represents tropomyosin-specific IgE. **A,** ROC curve for diagnosing persistent shrimp allergy, defined by a positive OFC. The AUC was 0.768 for shrimp-specific IgE and 0.683 for tropomyosin-specific IgE. **B,** ROC curve for shrimp allergy, including mild cases, in which mild allergy is defined as a negative OFC followed by recurrent symptoms upon home ingestion. The AUC was 0.741 for shrimp-specific IgE and 0.672 for tropomyosin-specific IgE.

In a subgroup analysis of patients with persistent shrimp allergy (n = 16) and tolerant patients (n = 40), ROC-curve analysis determined optimal cutoff values of 58.2 IUA/mL (sensitivity, 68.8%; specificity, 83.8%) for shrimp-specific IgE and 33.5 IUA/mL (sensitivity, 68.8%; specificity, 75.7%) for tropomyosin-specific IgE, with respective AUC values of 0.768 and 0.683 (Fig 1; Table IV). In a separate subgroup of 24 patients with either persistent or mild allergy and 40 tolerant patients, the optimal IgE cutoff values were 19.0 IUA/mL (sensitivity, 79.2%; specificity, 71.1%) for shrimp-specific IgE and 24.3 IUA/mL (sensitivity, 70.8%; specificity, 71.1%) for tropomyosin-specific IgE, with AUC values of 0.741 and 0.672, respectively, indicating higher diagnostic performance for shrimp-specific IgE.

**Table IV:** Optical cutoff values by ROC curves of shrimp- and tropomyosin-specific IgE levels

Category	Specific IgE	AUC	Cutoff (IUA/mL)	Sensitivity (%)	Specificity (%)
Persistent/tolerant∗	Shrimp	0.768	58.2	68.8	83.8
	Tropomyosin	0.683	33.5	68.8	75.7
Persistent and mild allergic/tolerant†	Shrimp	0.741	19.0	79.2	71.1
	Tropomyosin	0.672	24.3	70.8	71.1
Persistent/tolerant with past history‡	Shrimp	0.706	23.2	61.5	82.6
	Tropomyosin	0.622	13.9	61.5	69.6
Persistent and mild allergic/tolerant with past history§	Shrimp	0.744	19.8	73.7	82.6
	Tropomyosin	0.664	24.3	63.2	78.3

∗ Analysis of patients with persistent shrimp allergy (n = 16) and tolerant patients (n = 40).

† Analysis of patients with persistent and mild allergic shrimp allergy (n = 24) and tolerant patients (n = 40).

‡ Analysis of patients with symptomatic histories: persistent shrimp allergy (n = 13) and tolerant (n = 23).

§ Analysis of patients with symptomatic histories: persistent and mild allergic shrimp allergy (n = 19) and tolerant (n = 23).

ROC analysis was conducted on a subset of 43 patients with symptomatic histories (persistent: n = 13; mild allergic: n = 6; tolerant: n = 24), confirming the high optimal cutoff values in this subgroup (Table IV; see also Fig E2 in this article’s Online Repository at www.jaci-global.org). However, diagnostic accuracy did not improve.

The positive probability curve for OFC outcomes based on shrimp- and tropomyosin-specific IgE levels (see Fig E3 in this article’s Online Repository at www.jaci-global.org) indicated a 50% probability of a positive OFC at 114 IUA/mL for shrimp-specific IgE and at 173 IUA/mL for tropomyosin-specific IgE in persistent shrimp allergy. For persistent and mild allergy, these 50% probabilities were observed at 81 IUA/mL for shrimp-specific IgE and 119 IUA/mL for tropomyosin-specific IgE.

### SPT analysis

The median SPT wheal diameter for shrimp was 8.5 mm in patients with OFC-positive persistent allergy (n = 16), 9.5 mm in patients with mild allergy (n = 8), and 8.0 mm in tolerant patients (n = 40) (Table Ⅱ).

## Discussion

This study evaluated Japanese children with suspected shrimp allergy, confirming allergy status through OFC and assessing the diagnostic utility of shrimp-specific IgE and SPT. Generally, limited data exist on optimal cutoff values for shrimp- and tropomyosin-specific IgE in shrimp allergy diagnosis. Our findings identified optimal thresholds of 58.2 and 33.5 IUA/mL for shrimp- and tropomyosin-specific IgE, respectively, on the basis of ROC-curve analyses. These updated thresholds can assist clinicians in establishing more accurate diagnoses for shrimp allergy.

The optimal cutoff values for shrimp- and tropomyosin-specific IgE suggest that shrimp-specific IgE provides greater diagnostic accuracy than tropomyosin-specific IgE. We considered the possibility that house dust mite–specific IgE levels may have influenced the cutoff value. Similarly, Yamamoto-Hanada et al^10^ reported high IgE sensitization rates to *D farinae* in Japanese children, with mite-specific IgE (either *D pteronyssinus* or *D farinae*) exceeding 0.35 IUA/mL in all participants. Tuano et al^11^ reported shrimp-specific IgE as a more reliable marker than tropomyosin-specific IgE in patients without house dust mite sensitization, with cutoffs of 3.55 kUA/L (sensitivity, 100%; specificity, 85.7%) for those without house dust mite sensitization and 7.85 kUA/L (sensitivity, 57.1%; specificity, 54.5%) for those sensitized to mites. Furthermore, the cutoff value in this study was notably high. We believe cross-reactivity between shrimp and mite allergens likely contributed to elevated tropomyosin-specific IgE levels, even among OFC-negative patients in our study. Tagami et al^12^ also reported a strong correlation between mite and shrimp tropomyosins (Der p 10 and Pen a1) in Japanese children who underwent OFCs, regardless of shrimp allergy status. Among our study participants, 23 (35%) had not previously ingested shrimp, likely because of anxiety from high comorbidity with other food allergies (89%). To refine cutoff values, ROC analysis was conducted on a subset of 43 patients with symptomatic histories, showing that the high optical cutoff values observed in these patients were similar to those in patients without past histories.

However, further investigation is warranted into shrimp allergens closely associated with allergy symptoms in Japanese children, because the allergenic components of shrimp differ by region. Wai et al^13^ reported that troponin C (Pen m 6, 47.1%), glycogen phosphorylase (Pen m 14, 47.1%), tropomyosin (Pen m 1, 41.2%), and sarcoplasmic calcium-binding protein (Pen m 4, 35.3%) were significant shrimp allergens in Hong Kong, whereas Pen m 1 (68.8%), Pen m 6 (50.0%), and fatty acid–binding protein (Pen m 13, 37.5%) were more predominant in Thailand, highlighting such regional variations.

In this study, oral symptoms were the most common (69%), followed by gastrointestinal (56%), cutaneous (50%), and respiratory (44%) symptoms. Although most cases were mild, a 15-year-old developed anaphylaxis after consuming 2 g of shrimp. Chokshi et al^2^ also noted an anaphylaxis rate of 10% among children with shrimp allergies and 42% among adults, underscoring the importance of recognizing the risk of severe reactions, particularly in adolescents.

In addition, 8 patients (16%) in the OFC-negative group experienced mild recurrent symptoms after consuming shrimp, mirroring the common pattern of oral symptoms reported in 87.1% of patients allergic to shrimp by Thalayasingam et al.^14^

Notably, a single OFC may not conclusively confirm shrimp allergy in cases with low symptom scores (1-9 points). Miura et al^15^ reported improved diagnostic accuracy with repeated home-based ingestion in cases with uncertain symptoms for other food allergens (including egg, milk, and wheat), wherein 79.7% of uncertain cases were ultimately classified as nonallergic and 20.3% as allergic. Some children with low symptom scores appeared to have outgrown their clinical reactivity.

For OFC-negative patients, allergy or tolerance status was reassessed within 3 months on the basis of outpatient follow-up after home shrimp consumption. We speculate that inconclusive OFC results in this subgroup may be due to insufficient challenge doses. Therefore, we considered it necessary to instruct patients to gradually increase the amount of shrimp consumed at home, starting with a quantity that did not exceed the total OFC dose, to minimize the risk of symptoms. Although shrimp is a known trigger of food-dependent exercise-induced anaphylaxis (FDEIA), no such cases were reported among our OFC-negative participants. However, Harada et al^16^ documented shrimp as a major FDEIA trigger among Japanese adolescents, emphasizing the importance of FDEIA monitoring in adolescents, even for those with negative OFC results.

Furthermore, the SPT did not reliably predict symptom presence, because the most tolerant case of SPT was positive (median wheal diameter, 8.0 mm), suggesting that SPT results alone may not correlate with clinical symptoms.

This study had some limitations, including its relatively small sample size (67 participants), which may affect the generalizability of the findings. In addition, cross-reactivity between shrimp and house dust mite allergens could reduce test specificity, potentially affecting the accuracy of shrimp allergy diagnoses. Finally, the limited sample size may also have constrained the statistical power to detect subtle intergroup differences.

Shrimp SPT showed limited correlation with symptom onset. Tropomyosin-specific IgE and SPT were not superior to shrimp-specific IgE for diagnosing shrimp allergy in children with house dust mite–specific IgE. No IgE cutoff accurately predicts a successfully passed OFC.

**Clinical implications**

SPT showed limited symptom correlation, with shrimp-specific IgE being more diagnostic than tropomyosin-specific IgE. No IgE cutoff predicts a passed OFC, so diagnosis should not rely solely on these levels.

## Disclosure statement

Disclosure of potential conflict of interest: The authors declare that they have no relevant conflicts of interest.
