# Supplementary material for: Assessing diagnostic tests for shrimp allergy in children: A multicenter trial
Source: J Allergy Clin Immunol Glob. 2025 Apr 10;4(3):100471. doi: 10.1016/j.jacig.2025.100471 (PMC12053699; doi:10.1016/j.jacig.2025.100471)
Supplement: Supplementary Figs and Tables [file mmc1.docx]

**Supplementary Materials**


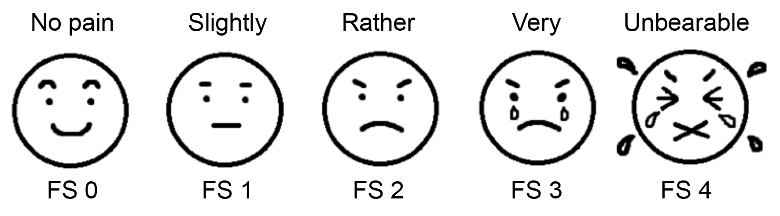


**Supplementary Figure 1.** Abdominal face pain scale (FS). The degree of abdominal pain is measured by the Face pain scale (FS).


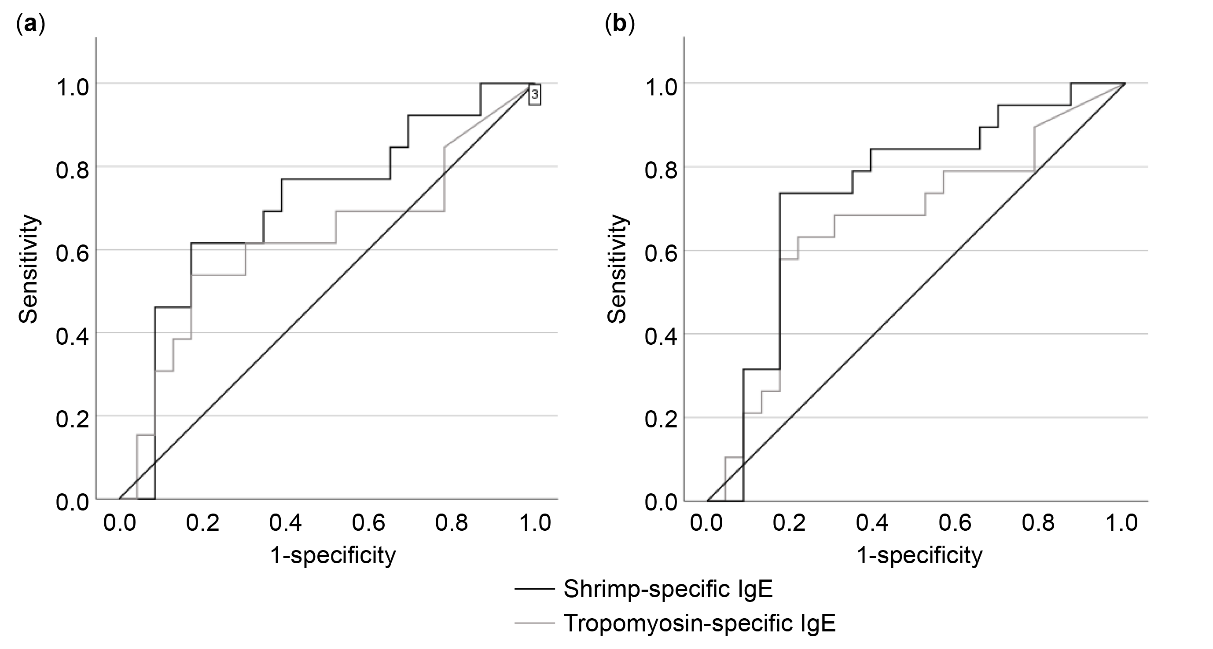


**Supplementary Figure 2.** Receiver operating characteristic (ROC) curves of shrimp- and tropomyosin-specific IgE on a subset of 43 patients with symptomatic histories. The black line represents shrimp-specific IgE, and the gray line represents tropomyosin-specific IgE. A: ROC curve for persistent shrimp allergy (n = 13), defined by a positive oral food challenge (OFC). The area under the curve (AUC) was 0.699 for shrimp-specific IgE and 0.622 for tropomyosin-specific IgE. B: ROC curve for shrimp allergy, including mild cases (n = 19), where mild allergy is defined as a negative OFC followed by recurrent symptoms upon home ingestion. The AUC was 0.737 for shrimp-specific IgE and 0.663 for tropomyosin-specific IgE. Abbreviations: ROC, receiver operating characteristic; IgE, immunoglobulin E

**
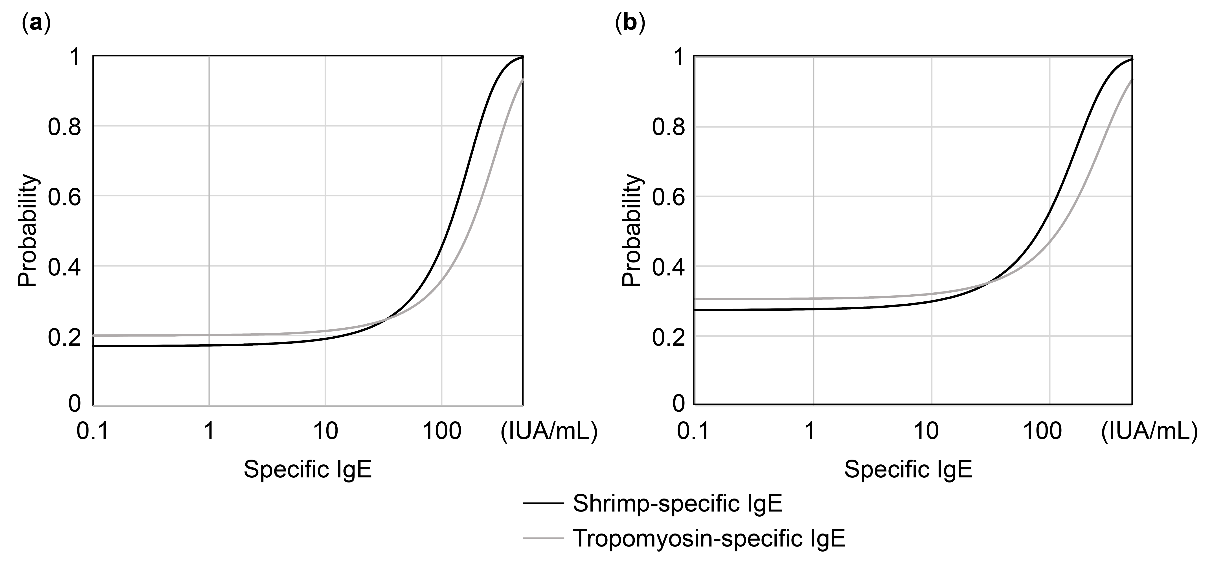
**

**Supplementary Figure 3.** Probability curves of shrimp- and tropomyosin-specific IgE. The black line represents shrimp-specific IgE, and the gray line represents tropomyosin-specific IgE. A: Probability curve for persistent shrimp allergy defined by a positive oral food challenge (OFC). B: Probability curve for shrimp allergy, including mild cases, where mild allergy is defined as a negative OFC followed by recurrent symptoms upon home ingestion.

Abbreviations: IgE, immunoglobulin E

**Supplementary Table Ⅰ**. Anaphylaxis scoring system Aichi (ASCA)

| Score organ | 0 | ①１point | ① 5 points | ② 10 points | ② 20 points | ➂ 40 points | ④ 60 points |
| --- | --- | --- | --- | --- | --- | --- | --- |
| Respiratory | None | Itchy nose | Laryngeal discomfort | Nasal congestion | Speech disturbance | Loss of voice |  |
| *(subjective)* |  |  |  | Suffocating breath | Difficulty in breathing |  |  |
| *(objective)* |  | Sneeze | Mild transient coughing | Intermittent coughing | Frequent coughing | Continuous coughing | Weak breath sounds |
|  |  |  | Runny nose | Mild wheezing | Apparent wheezing | Strong wheezing | Strong retraction |
|  |  |  |  |  | Hoarseness | Intentional breathing | Cyanosis |
|  |  |  |  |  |  | Inspiratory strider | SpO_2_ ≤ 90% |
|  |  |  |  |  |  | Retraction |  |
| Skin/Mucosal | None | Itch (around mouth) | Itch (local and mild) | Itch (whole body) | Unbearable itch |  |  |
| *(subjective)* |  | Mild discomfort, |  |  |  |  |  |
|  |  | Burning sensation |  |  |  |  |  |
| *(objective)* |  | <*Peri-oral*> | <*Local*> | <*Multiple*> | *<Spreading,Generalized>* | |  |
|  |  | Hives, Erythema, Swelling, Vesicle | Eye edema, Bloodshot, Hives, Erythema, Swelling, Angioedema | Hives, Erythema, Swelling, Angioedema | Hives, Erythema, Swelling, Angioedema |  |  |
| Gastrointestinal | None | Oral or pharyngeal itch, Hot taste | Mild nausea, | Mild nausea. | Strong abdominal pain (FS3) | Unbearable abdominal pain (FS4) | |
| *(subjective)* |  |  | Abdominal pain (FS1) | Abdominal pain |  |  |  |
| *(objective)* |  |  | Increased bowel sounds | Diarrhea, Vomiting | Recurrent Vomiting | Dehydration by vomiting | |
| Psychoneurological | None | Refusal to eat | Loss of activity | Sleep, Tendency to lay down | Sleep (not usual) | Tend to fall unconscious | Unconsciousness |
|  |  | Mild excitement | Irritation | Mild excitement | Agitating. Crying | Uncontrolled panic |  |
| Cardiovascular | None |  |  |  |  | Pale, Tachycardia | Bradycardia |
|  |  |  |  |  |  | Cold extremities |  |
|  |  |  |  |  |  | Cold sweat |  |
|  |  |  |  |  |  | Mild decrease in blood pressure | Low Blood Pressure |
| *(Blood pressure)* |  |  |  |  |  | <1 y;<70 mmHg | <1 y; <50 mmHg |
|  |  |  |  |  |  | 1–10 y; 70+ | 1–10 y; 60+ |
|  |  |  |  |  |  | (2×age) mmHg | (2×age) mmHg |
|  |  |  |  |  |  | 11–17 y; <90 | 11–17 y; <70 mmHg |

Each organ symptom score (0–60 points) is given based on the severity of the problem, which is defined as a sum of the highest organ symptom score observed throughout the source of allergic symptoms (maximum 240 points). FS; Face pain scale to express the degree of abdominal pain (Supplementary Figure 1)
